# FLAVOUR Study: FLow profiles And postoperative VasOplegia after continUous-flow left ventriculaR assist device implantation

**DOI:** 10.1007/s12265-023-10476-5

**Published:** 2024-02-01

**Authors:** Bas J. Kersten, Lieke Numan, Marnix M. van der Schoot, Michel de Jong, Faiz Ramjankhan, Emmeke Aarts, Marish I. F. J. Oerlemans, Linda W. van Laake, Eric E. C. de Waal

**Affiliations:** 1 Department of Anesthesiology, University Medical Center Utrecht, Post Office Box 85500, 3508 Utrecht, GA Netherlands; 2 Department of Cardiology, University Medical Center Utrecht, Utrecht, Netherlands; 3 Department of Anesthesiology, Leiden University Medical Center, Leiden, Netherlands; 4 Heartbeat Perfusion, University Medical Center Utrecht, Utrecht, Netherlands; 5 Department of Cardiothoracic Surgery, University Medical Center Utrecht, Utrecht, Netherlands; 6 Department of Methodology and Statistics, Utrecht University, Utrecht, Netherlands

**Keywords:** Cardiac vasoplegia syndrome, Continuous-flow left ventricular assist device, Flow profile, Axial flow, Centrifugal flow, Centrifugal flow with artificial pulse, Outcomes, Morbidity, Mortality

## Abstract

**Abstract:**

This study aims to associate the incidence of postoperative vasoplegia and short-term survival to the implantation of various left ventricular assist devices differing in hemocompatibility and flow profiles. The overall incidence of vasoplegia was 25.3% (73/289 patients) and 30.3% (37/122), 25.0% (18/72), and 18.9% (18/95) in the axial flow (AXF), centrifugal flow (CF), and centrifugal flow with artificial pulse (CFAP) group, respectively. Vasoplegia was associated with longer intensive care (ICU) and hospital length of stay (LOS) and mortality. ICU and in-hospital LOS and 1-year mortality were the lowest in the CFAP group. Post hoc analysis resulted in a *p*-value of 0.43 between AXF and CF; 0.35 between CF and CFAP; and 0.06 between AXF and CFAP. Although there is a trend in diminished incidence of vasoplegia, pooled logistic regression using flow profile and variables that remained after feature selection showed that flow profile was not an independent predictor for postoperative vasoplegia.

**Graphical Abstract:**

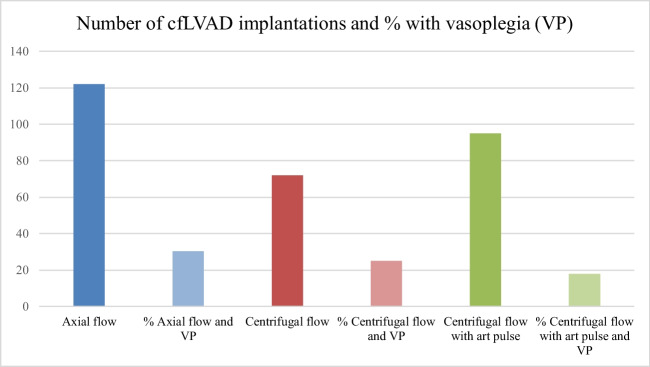

**Supplementary Information:**

The online version contains supplementary material available at 10.1007/s12265-023-10476-5.

## Background

Left ventricular assist devices (LVADs) are increasingly used in the treatment of end-stage heart failure as a bridge to heart transplantation, destination therapy, or bridge to recovery. Compared to medical heart failure treatment, mechanical circulatory support (MCS) improves survival and quality of life [1, 2]. Moreover, long-term outcomes further improved due to better preoperative patient selection, improved intraoperative care, and improved device design [3–7]. Recent available continuous-flow LVADs (cfLVADs) differ in internal characteristics, such as hemocompatibility and flow profiles: from axial flow to centrifugal flow to centrifugal flow with artificial pulse, with the latter increasing the level of pulsatility even producing (sub)physiologic pulse pressures [8–11].

It is shown that continuous flow results in an increased shear stress on the endothelial cells resulting in increased endothelial permeability, increased nitric oxide (NO) formation in the endothelial cell, diminished actin–myosin contraction and more vasodilation [12, 13]. As the human circulation is pulsatile, pulsatile flow profiles have an opposite effect on shear stress and thus less endothelial permeability, less NO formation and a reduction in the diminished actin–myosin contraction, and finally less vasodilation.

Vasoplegia is a common immediate postoperative complication after LVAD implantation characterized by severe hypotension unresponsive to vasopressor therapy due to a loss in vasomotor tone [14–17]. Different flow profiles of the various assist devices may play a role in this vasodilatory state of the vessels. However, the exact pathophysiological mechanisms in the development of vasoplegia are still unknown. This is clinically highly relevant, since vasoplegia may affect early postoperative outcomes and survival [15–17]. Recent literature on the incidence of immediate postoperative vasoplegia associated with different cfLVAD types with unique flow profiles is lacking.

The goal of this study is to investigate whether there is a difference in incidence of vasoplegia after implantation of different flow profile cfLVADs and to explore if there is a difference in short-term outcomes related to vasoplegia and different flow profile cfLVADs.

## Methods

### Study Population

This study was designed as a single-center retrospective analysis. All procedures followed were in accordance with the ethical standards of the responsible committee on human experimentation and conducted following Good Clinical Practice and the 2002 Declaration of Helsinki. The study was approved by the local ethics committee with an exemption from requiring ethical approval (METC: 20-110/c) as patients were not subjected to any investigational action. Adult patients 18 years and older, implanted with a cfLVAD in January 2006 through December 2020, were included. Since this study focused on the effect of flow profiles after primary implantation, patients with a preoperatively implanted assist devices (cfLVAD, intra-aortic balloon pump, ECMO, Impella, etc.) or with intraoperative right ventricular failure necessitating (temporary) right ventricular assist device support (RVAD) were excluded.

### Perioperative Monitoring

All patients were perioperatively monitored using basic hemodynamic monitoring (ECG, SaO_2_, and non-invasive blood pressure) and advanced hemodynamic monitoring using a radial arterial line, a pulmonary artery catheter, and intraoperative TEE.

### Primary Endpoint

The primary outcome was the incidence of vasoplegia in the different flow profile groups: axial flow (HeartMate II, HMII), centrifugal flow (HVAD), and centrifugal flow with artificial pulse (HeartMate 3, HM3). We used the unified definition of vasoplegia as described previously: mean arterial pressure (MAP) ≤ 50 mmHg and/or systemic vascular resistance (SVR) ≤ 800 dynes∙s∙cm^−5^ (vasodilation criterion); cardiac index (CI) ≥ 2.5 l∙min^−1^∙m^−2^ (hemodynamic criterion); use of norepinephrine ≥ 200 ng∙kg^−1^∙min; or equivalent doses of vasopressors conform ATHOS-3 trial (epinephrine ≥ 200 ng∙kg^−1^∙min^−1^; dopamine ≥ 30 μg∙kg^−1^∙min; phenylephrine ≥ 2 μg∙kg^−1^∙min, or vasopressin ≥ 0.08 U∙min^−1^ (high vasopressor requirement), for at least 3 consecutive hours (time criterion) in the first 48 h after arrival at the ICU [16, 18].

### Secondary Endpoints

The secondary endpoints were incidence of serious immediate adverse perioperative events, such as development of tamponade, major bleeding, renal failure, right ventricular failure, prolonged postoperative mechanical ventilation, re-intubation, and long-term outcomes [19].

### Data Collection

Pre- and postoperative data were extracted from the electronic hospital information system (HiX, ChipSoft, Amsterdam, Netherlands) and intensive care unit (ICU) data monitoring system (Metavision, iMDsoft, Düsseldorf, Germany). Preoperative RV function was scored by two independent cardiologists as poor, intermediate, or good based on echocardiography and right heart catheterization data (right atrial pressures, pulmonary artery pressures, pulmonary capillary wedge pressure, cardiac index, right ventricular stroke work index) [4]. The intraoperative data were extracted from our anesthesia information system (Anstat, Carepoint, Ede, Netherlands).

### Statistical Analysis

Statistical analysis was performed using SPSS version 24 for Mac (SPSS Inc, Chicago, IL, USA) and R version 3.6.3 (Vienna, Austria). Continuous data were presented as mean ± standard deviation (SD) or median (interquartile range), while categorical data were presented as counts and percentages. Missing data were presented as percentages of missing data per variable. For continuous variables, a *t*-test was used for the comparison of two groups. For more than 2 groups, a one-way ANOVA was used with Tukey’s method for post hoc analysis. For categorical variables, a chi-square analysis was used. To evaluate the effect of flow profile on vasoplegia, we combined multiple imputation and lasso regression. At first, multiple imputation was used to impute missing data, resulting in ten imputed datasets (R package: mice) [20]. Subsequently, within all ten imputed datasets, we performed logistic regression with least absolute shrinkage and selection operator (LASSO) using all collected variables (Table 1). Logistic LASSO regression was used to prevent overfitting and to obtain an interpretable set of variables to use in a multivariate logistic regression model together with the variable of interest (flow profile). Fivefold cross-validation was performed to obtain the optimal regularization parameter (lambda) used in LASSO. Variables that remained non-zero after lasso regression with the optimal lambda in all ten imputed datasets were selected for the final logistic regression model in the imputed datasets. Results were pooled over the imputed datasets to evaluate the effect of device type on the incidence of vasoplegia adjusted for the selected variables. A *p*-value of <0.05 was assumed to be significant.

**Table 1:** Baseline characteristics stratified to LVAD flow profiles

	Axial flow (*n*=122)	Centrifugal flow (*n*=72)	Centrifugal flow with artificial pulse (*n*=95)	*p*-value
Demographic data				
Age (years)	49.2 ± 12.8	54.6 ± 12.8	52.4 ± 13.6	0.02
Male gender	85 (69.7%)	45 (62.5%)	63 (66.3%)	0.59
Weight (kg)	74.3 ± 14.2	74.4 ± 13.6	78.0 ± 14.5	0.13
BSA (m^2^)	1.91 ± 0.20	1.90 ± 0.19	1.95 ± 0.20	0.12
BMI (kg/m^2^)	23.7 ± 4.0	24.1 ± 3.9	24.6 ± 4.0	0.28
Patient history				
Systolic blood pressure (mmHg)	99 ± 15	103 ± 15	102 ± 13	0.19
Diastolic blood pressure (mmHg)	63 ± 11	68 ± 12	65 ± 11	0.02
Smoking history	60 (49.2%)	23 (31.9%)	40 (42.1%)	0.06
COPD	17 (13.9%)	13 (18.1%)	9 (9.5%)	0.27
Previous cardiothoracic surgery	18 (14.8%)	12 (16.7%)	11 (11.6%)	0.63
Preoperative medication				
LMWH	24 (19.7%)	28 (38.9%)	55 (57.9%)	<0.01
Beta blocker	42 (34.4%)	18 (25.0%)	17 (17.9%)	0.02
ACE inhibitor	52 (42.6%)	25 (34.7%)	19 (20.0%)	<0.01
ATII receptor blocker	15 (12.3%)	11 (15.3%)	26 (27.4%)	0.01
Loop diuretics	112 (91.8%)	63 (87.5%)	87 (91.6%)	0.57
Aldosterone antagonist	85 (69.7%)	59 (81.9%)	79 (83.2%)	0.03
Dopamine	23 (18.9%)	2 (2.8%)	4 (4.2%)	n.s.
Dobutamine	74 (60.7%)	35 (48.6%)	45 (47.4%)	0.10
Milrinone	52 (42.6%)	26 (36.1%)	35 (36.8%)	0.57
Noradrenaline	4 (3.3%)	4 (5.6%)	7 (7.4%)	n.s.
Preoperative laboratory data				
Hemoglobin (mmol/L)	7.7 ± 1.2	8.2 ± 1.2	8.3 ± 1.2	<0.01
*Bilirubin* (*umol/L*)	*27 [18–40]*	*20 [12–32]*	*18 [11–32]*	*<0.01*
*ASAT* (*U/L*)	*36 [27–69]*	*34 [25–56]*	*34 [22–49]*	*0.32*
*ALAT* (*U/L*)	*49 [27–121]*	*40 [26–64]*	*41 [25–105]*	*0.07*
Creatinine (umol/L)	124 ± 44	121 ± 55	115 ± 44	0.32
GFR (mL/min)	61 ± 23	63 ± 27	68 ± 29	0.20
Type of heart failure				
Ischemic	24 (19.7%)	23 (31.9%)	20 (21.1%)	0.15
Dilated	79 (64.8%)	44 (61.1%)	70 (73.7%)	0.16
Myocarditis	6 (4.9%)	1 (1.4%)	1 (1.1%)	n.s.
Peripartum	3 (2.5%)	1 (1.4%)	1 (1.1%)	n.s.
Hypertrophic	4 (3.3%)	2 (2.8%)	1 (1.1%)	n.s.
Toxic	6 (4.9%)	1 (1.4%)	2 (2.2%)	n.s.
Right ventricular function				
Bad	24 (20.2%)	7 (9.7%)	12 (12.6%)	0.11
Moderate	73 (61.3%)	42 (58.3%)	48 (50.5%)	0.27
Good	22 (18.5%)	23 (31.9%)	35 (36.8%)	0.01
INTERMACS classification				
INTERMACS class I	4 (3.3%)	3 (4.2%)	4 (4.2%)	0.92
INTERMACS class II	67 (54.9%)	24 (33.3%)	34 (35.8%)	<0.01
INTERMACS class III	43 (35.2%)	29 (40.3%)	33 (34.7%)	0.72
INTERMACS class IV–VI	8 (6.6%)	16 (22.2%)	24 (25.3%)	<0.01

Values are mean ± standard deviation or numbers with percentages. *ACE* angiotensin-converting enzyme. *ALAT* alanine transaminase. *ASAT* aspartate transaminase. *ATII* angiotensin II. *BMI* body mass index. *BSA* body surface area. *CARA* chronic aspecific respiratory disease. *COPD* chronic obstructive pulmonary disease. *GFR* glomerular filtration rate. *LMWH* low molecular weight heparin. *n.s.* no statistics performed (for example, because of too little numbers)

## Results

### Patients

**Fig. 1:**
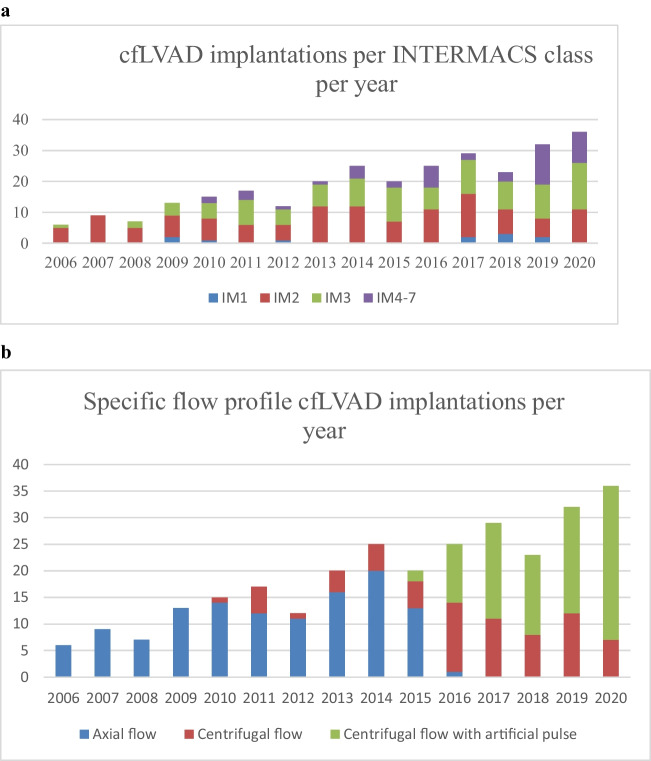
Number of LVAD implantations per INTERMACS classification per year (**a**) and specific flow pattern LVAD implantations (**b**). IM, INTERMACS

In 393 patients, a cfLVAD was implanted in January 2006 through December 2020. Of these, 289 patients received a primary long-term cfLVAD. Baseline characteristics, such as demographic data, patient history, preoperative medication, preoperative laboratory data, type of heart failure, preoperative right ventricular function, and Interagency Registry for Mechanically Assisted Circulatory Support (INTERMACS) data, are presented in Table 1. Patients in the axial flow group received more often angiotensin-converting enzyme inhibitors than patients with centrifugal flow with/without artificial pulse but less angiotensin II receptor blockers. Patients in the axial flow group had lower preoperative hemoglobin (Hb) and higher bilirubin levels compared to centrifugal flow and centrifugal flow with artificial pulse. INTERMACS scores of patients included and flow profiles per year and number of patients with vasoplegia are presented in Figure 1a and b, respectively. There is a significant difference in the INTERMACS score in the axial flow group (58.2% INTERMACS class I or II) compared to the centrifugal flow group (37.5% INTERMACS class I or II) and centrifugal flow with artificial pulse group (40.0% INTERMACS class I or II) (*p*=0.003). In addition, 6.6% of the patients implanted with an axial flow were classified as INTERMACS class IV–VI, while 22.2% and 25.3% of the centrifugal flow group and centrifugal flow with artificial pulse group, respectively, were classified as INTERMACS IV–VI (*p*<0.01). Baseline characteristics stratified to vasoplegia are presented in Table 2. Moreover, baseline characteristics stratified to flow profiles and postoperative vasoplegia are presented in supplemental tables 2a, 2b, and 2c. Although several baseline characteristics were significant between patients with and without vasoplegia (Table 2), there is a trend in differences when stratifying these patients to specific cfLVAD flow profiles (supplemental tables 2a, 2b and 2c).

**Table 2:** Baseline characteristics of patients stratified to vasoplegia

	Vasoplegia (*n*=73)	No vasoplegia (*n*=216)	*p*-value
Demographic data			
Age (years)	54.0 ± 11.7	50.8 ± 13.6	0.08
Male gender	59 (80.8%)	134 (62.0%)	<0.01
Weight (kg)	77.0 ± 13.6	75.0 ± 14.4	0.30
BSA (m^2^)	1.95 ± 0.18	1.91 ± 0.20	0.97
BMI (kg/m^2^)	24.1 ± 3.5	24.1 ± 4.1	0.14
Patient history			
Systolic blood pressure (mmHg)	97 ± 15	102 ± 14	<0.01
Diastolic blood pressure (mmHg)	62 ± 10	66 ± 11	0.03
Smoking history	34 (46.6%)	89 (41.2%)	0.42
COPD/CARA	6 (8.2%)	33 (15.3%)	0.13
Previous cardiothoracic surgery	20 (27.4%)	21 (9.7%)	<0.01
Preoperative medication			
LMWH	19 (20.6%)	88 (40.7%)	0.02
Beta blocker	18 (24.7%)	59 (27.3%)	0.66
ACE inhibitor	17 (23.3%)	79 (36.6%)	0.04
ATII receptor antagonist	19 (26.0%)	33 (15.3%)	0.01
Lis-diuretics	69 (94.5%)	193 (89.4%)	0.19
Aldosterone antagonist	52 (71.2%)	171 (79.2%)	0.16
Dopamine	12 (16.4%)	17 (7.9%)	0.04
Dobutamine	41 (56.2%)	113 (52.3%)	0.57
Milrinone	32 (43.8%)	81 (37.5%)	0.34
Noradrenaline	6 (8.2%)	9 (4.2%)	0.18
Preoperative laboratory data			
Hemoglobin (mmol/L)	7.7 ± 1.2	8.1 ± 1.2	0.02
*Bilirubin* (*umol/L*)	*32 [23–43]*	*23 [16–38]*	*<0.01*
* ASAT* (*U/L*)	*36 [27–85]*	*36 [26–66]*	*0.53*
*ALAT* (*U/L*)	*44 [26–163]*	*49 [27–105]*	*0.97*
Creatinine (umol/L)	149 ± 59	111 ± 38	<0.01
GFR (mL/min)	52 ± 23	68 ± 26	<0.01
Type of heart failure			
Ischemic	27 (37.0%)	40 (18.5%)	<0.01
Dilated	42 (57.5%)	151 (69.9%)	0.04
Myocarditis	0 (0.0%)	8 (3.7%)	n.s.
Peripartum	0 (0.0%)	5 (2.3%)	n.s.
Hypertrophic	3 (4.1%)	4 (1.9%)	n.s.
Toxic	1 (1.4%)	8 (3.7%)	n.s.
Right ventricular function			
Bad	10 (13.7%)	33 (15.3%)	0.75
Moderate	46 (63.0%)	117 (54.2%)	0.17
Good	16 (21.9%)	64 (29.6%)	0.21
INTERMACS classification			
INTERMACS class I	4 (5.5%)	7 (3.2%)	0.37
INTERMACS class II	35 (47.9%)	90 (41.7%)	0.60
INTERMACS class III	24 (32.9%)	81 (37.5%)	0.57
INTERMACS class IV–VI	10 (13.7%)	38 (17.6%)	0.64

Values are mean ± standard deviation or numbers with percentages. *ACE* angiotensin-converting enzyme. *ALAT* alanine transaminase. *ASAT* aspartate transaminase. *ATII* angiotensin II. *BMI* body mass index. *BSA* body surface area. *CARA* chronic aspecific respiratory disease. *COPD* chronic obstructive pulmonary disease. *GFR* glomerular filtration rate. *LMWH* low molecular weight heparin. *n.s.* no statistics performed (for example, because of too little numbers)

Intraoperative data stratified to cfLVAD flow profiles are presented in Table 3. Intraoperative transfusion of blood products was higher in the axial flow group compared to the centrifugal flow group and the centrifugal flow with artificial pulse group for red blood cells (RBC) fresh frozen plasma units (FFP) and total units of blood products. Intraoperative data stratified to vasoplegia are presented in Table 4.

**Table 3:** Intraoperative data stratified to cfLVAD flow profiles generated by the cfLVAD

	Axial flow (*n*=122)	Centrifugal flow (*n*=72)	Centrifugal flow with artificial pulse (*n*=95)	*P*-value
Skin-to-skin time (min)	232 [200–288]	234 [197–285]	226 [196–259]	0.45
Bypass time (min)	107 [91–129]	107 [85–149]	111 [93–130]	0.87
Red blood cells (units)	0 [0–2]	0 [0–2]	0 [0–0]	0.01
Thrombocytes (units)	0 [0–1]	1 [0–1]	0 [0–1]	0.40
Fresh Frozen Plasma (units)	2 [0–4]	2 [0–3]	2 [0–2]	0.12
Total number of units of blood	2.5 [0–5]	3 [0–5]	2 [0–4]	0.05
Cell Saver (ml)	480 [200–748]	480 [200–748]	500 [400–750]	0.06
Milrinone	116 (95.1%)	63 (87.5%)	86 (90.5%)	0.16
Dobutamine	110 (90.2%)	68 (94.4%)	81 (85.3%)	0.15
Noradrenaline	117 (95.9%)	70 (97.2%)	95 (100%)	0.15
Dopamine	26 (21.3%)	1 (1.4%)	3 (3.2%)	n.s.
Nitric Oxide	102 (83.6%)	59 (81.9%)	67 (70.5%)	< 0.05

Values are mean ± standard deviation or numbers with percentages. n.s. no statistics performed (for example, because of too little numbers)

**Table 4:** Intraoperative data stratified to postoperative vasoplegia

	Vasoplegia (*n*=73)	No Vasoplegia (*n*=216)	*P*-value
Skin-to-skin time (min)	256 [198–317]	230 [199–266]	0.01
Bypass time (min)	118 [93–148]	107 [89–129]	0.03
Red blood cells (units)	0 [0–2]	0 [0–1]	0.04
Thrombocytes (units)	1 [0–1]	0 [0–1]	< 0.01
Fresh Frozen Plasma (units)	2 [0–4]	1 [0–2]	< 0.01
Total units of blood products	4 [2–7]	2 [0–4]	< 0.01
Cell Saver (mL)	500 [300–975]	500 [200–700]	0.09
Milrinone	68 (93.2%)	197 (91.2%)	0.60
Dobutamine	64 (87.7%)	195 (90.3%)	0.52
Noradrenaline	70 (95.9%)	212 (98.1%)	0.28
Dopamine	12 (16.4%)	18 (8.4%)	< 0.05
Nitric Oxide	60 (82.2%)	168 (77.8%)	0.42

Values are mean ± standard deviation or numbers with percentages, *n.s.* No statistics performed (for example because of too little numbers)

### Primary Outcome

Using the unified definition of vasoplegia, the incidence of vasoplegia in the complete cohort was 25.3% (73 out of 289 included patients). Of all patients with vasoplegia, the SVR criterion (< 800 dynes∙s∙cm^−5^) was positive in 67 patients (91.8%), the MAP criterion (< 50 mmHg) in one patient (1.4%), and both criteria (SVR < 800 dynes∙s∙cm^−5^ and MAP ≤ 50 mm Hg) in 5 patients (6.8%).

The incidence of postoperative vasoplegia between the three cfLVAD flow profiles was 30.3% (37/122) in the axial flow group; 25.0% (18/73) in the centrifugal flow group; and 18.9% (18/95) in the centrifugal flow with artificial flow group with *p* = 0.16. Moreover, a post hoc analysis was performed, using Tukey’s method, resulting in a *p*-value of 0.43 between axial and centrifugal flow; a *p*-value of 0.35 between centrifugal flow with artificial pulse and centrifugal flow; and a *p*-value of 0.06 between axial flow and centrifugal flow with artificial pulse was found. Taken together, there was no significant difference in the first occurrence of vasoplegia in the respective cfLVAD flow groups.

### Secondary Outcomes

Postoperative use of inotropes and vasopressors are represented in Tables 3 and 4 and supplemental tables 3, 4, 5a, 5b, and 5c. Norepinephrine was used in a higher mean dosage in vasoplegic patients with an axial flow device, while vasopressin was used in a higher mean dosage in vasoplegic patients with a centrifugal flow with artificial pulse device. Moreover, ≥ 2 inotropes were more often used in patients with an axial flow device, while ≥ 2 vasopressors were more often used in patients with a centrifugal flow with artificial pulse device, regardless of the occurrence of vasoplegia.

Postoperative outcomes stratified to all patients with and without vasoplegia are presented in Table 5. Postoperative outcomes were worst in the vasoplegia group compared to the no vasoplegia group. Postoperative outcomes stratified to cfLVAD flow profiles and vasoplegia are presented in Table 6. The incidence of postoperative RV failure was higher in the overall vasoplegia group compared to the no vasoplegia group (*p*=0.01), higher in the vasoplegia group considering flow profiles and significantly higher in the axial flow profile group with vasoplegia. This difference was most distinctive in the group with a preoperative bad right ventricular function and postoperative vasoplegia, as shown in Table 7. Moreover, postoperative outcomes were better in the centrifugal flow with artificial pulse group than in the centrifugal flow group and even more superior when compared to the axial flow group.

**Table 5:** Postoperative outcomes in patients with and without postoperative vasoplegia

	Vasoplegia (*n*=73)	No vasoplegia (*n*=216)	*p*-value
Ventilation (hours)	80 [49–163]	45 [26–93]	< 0.01
Re-intubation	9 (12.3%	20 (9.3%)	0.45
Major bleeding	22 (30.1%)	28 (13.0%)	< 0.01
Tamponade	16 (22.5%)	26 (12.4%)	0.04
Renal failure	25 (34.2%)	30 (13.9%)	< 0.01
Right ventricular failure	31 (42.5%)	59 (27.3%)	0.01
ICU Length of stay (days)	6.4 [4.1–12.2]	4.9 [3.0–7.1]	< 0.01
ICU mortality	9 (12.3%)	10 (4.6%)	0.02
Hospital Length of stay (days)	27.5 [21.0–42.4]	26.4 [19.4–34.3]	0.02
Hospital mortality	10 (13.7%)	13 (6.0%)	0.04
30-day mortality	7 (9.6%)	8 (3.7%)	0.05
1-year mortality	17 (25.8%)	19 (10.2%)	< 0.01

Values are mean ± standard deviation or numbers with percentages. *ICU* intensive care unit

**Table 6:** Postoperative outcomes stratified to cfLVAD flow profiles and vasoplegia

	Axial flow			Centrifugal flow			Centrifugal flow with artificial pulse		
	Vasoplegia (*n*=37)	No vasoplegia (*n*=85)	*P*-value	Vasoplegia (*n*=18)	No Vasoplegia (*n*=54)	*P*-value	Vasoplegia (*n*=18)	No Vasoplegia (*n*=77)	*p*-value
Ventilation (hours)	135 [77–189]	47 [22–93]	< 0.01	71 [38–91]	50 [30−92]	0.43	51 [29−68]	39 [26−99]	0.49
Re-intubation	7 (18.9%)	4 (4.7%)	0.01	0 (0.0%)	4 (7.4%)	n.s.	2 (11.1%)	12 (15.6%)	n.s.
Major bleeding	16 (43.2%)	5 (5.9%)	< 0.01	4 (22.2%)	12 (22.2%)	n.s.	2 (11.1%)	11 (14.3%)	n.s.
Tamponade	11 (29.7%)	1 (1.2%)	n.s.	4 (22.2%)	12 (23.1%)	n.s.	1 (6.3%)	13 (17.8%)	n.s.
Renal failure	16 (43.3%)	10 (11.8%)	< 0.01	6 (33.3%)	8 (14.8%)	0.09	3 (16.7%)	12 (15.6%)	n.s.
Right ventricular failure	19 (51.4%)	17 (20.0%)	< 0.01	6 (33.3%)	17 (31.5%)	0.88	6 (33.3%)	25 (32.5%)	0.94
ICU Length of stay (days)	9.8 [6.1–19.6]	6.0 [3.9–7.9]	< 0.01	5.0 [4.8–6.7]	4.1 [2.9–7.2]	0.29	3.9 [3.0−6.8]	4.1 [2.9−6.8]	0.40
ICU mortality	6 (16.2%)	4 (4.7%)	0.07	3 (16.7%)	4 (7.4%)	n.s.	0 (0.0%)	2 (2.6%)	n.s.
Hospital Length of stay (days)	36.4 [23.3–64.6]	27.8 [19.9–35.9]	< 0.01	27.4 [21.3–33.3]	24.0 [19.4–31.6]	0.30	23.4 [20.1−26.9]	26.4 [19.4−33.4]	0.40
Hospital mortality	6 (16.2%)	7 (8.2%)	0.19	3 (16.7%)	4 (7.4%)	n.s.	1 (5.6%)	2 (2.6%)	n.s.
30-day mortality	4 (10.8%)	5 (5.9%)	n.s.	2 (11.1%)	2 (3.7%)	n.s.	1 (5.6%)	1 (1.2%)	n.s.
1-year mortality	10 (27.0%)	8 (8.4%)	0.01	6 (35.2%)	8 (17.0%)	0.12	1 (8.3%)	3 (5.6%)	n.s.

Values are mean ± standard deviation or numbers with percentages. *ICU* intensive care unit; *n.s*. no statistics performed (for example, because of too little numbers)

**Table 7:** Postoperative RV failure in relation to preoperative RV function and postoperative vasoplegia

Good preoperative RV function 80 patients	Vasoplegia (*n*=16)	No vasoplegia (*n*=64)	*p*-value
Postoperative RV-failure < 30 days	6 (37.5%)	14 (21.9%)	0.17
Moderate preoperative RV function 163 patients	Vasoplegia (*n*=46)	No vasoplegia (*n*=117)	*p*-value
Postoperative RV-failure < 30 days	17 (37.0%)	34 (29.1%)	0.21
Bad preoperative RV function 43 patients	Vasoplegia (*n*=10)	No vasoplegia (*n*=33)	*p*-value
Postoperative RV-failure < 30 days	7 (70.0%)	11 (33.3%)	< 0.05

### LASSO Regression

A total of 10 variables were selected by LASSO regression (Table 8) with an optimal lambda ranging between 0.033 and 0.039. Pooled logistic regression showed that device type was not a significant independent predictor (axial flow, HR 1.45 [0.64–2.26]. *p*=0.37; centrifugal flow, HR 1.39 [0.50–2.26], *p*=0.47) compared to centrifugal flow with artificial pulse. Previous cardiothoracic surgery, preoperative use of angiotensin II receptor blockers, preoperative bilirubin and creatinine levels, and the perioperative use of fresh frozen plasma were independent predictors for vasoplegia.

**Table 8:** Multivariate logistic regression results using variables selected by LASSO in addition to cfLVAD flow profiles

Variable	Hazard Ratio (HR) [95% CI]	p-value
Axial flow versus centrifugal flow with artificial pulse	1.45 [0.64–2.26]	0.37
Centrifugal flow versus centrifugal flow with artificial pulse	1.39 [0.50–2.28]	0.47
Male gender	1.61 [0.85–2.38]	0.22
Systolic blood pressure (mmHg)	0.98 [0.95–1.00]	0.07
Previous cardiothoracic surgery	2.79 [1/93–3.65]	0.02
ACE-inhibitor	0.62 [0.00–1.39]	0.22
ATII-receptor blocker	2.89 [2.06–3.73]	0.01
Dopamine	1.91 [0.86–2.96]	0.23
Ischemic cardiomyopathy	1.55 [0.80–2.31]	0.25
Bilirubin (μmol/L)	1.02 [1.01–1.04]	0.02
Creatinine (μmol/L)	1.02 [1.01–1.02]	< 0.01
Fresh Frozen Plasma (units)	1.25 [1.09–1.41]	0.01

Values are hazard ratio with 95% confidence interval

## Discussion

To our knowledge, this is the first study reporting the incidence of postoperative vasoplegia associated with the implantation of different cfLVADs with their various effects on hemocompatibility and generating different flow profiles. Although there seems a tendency in a diminished incidence of vasoplegia in the centrifugal flow with artificial pulse group compared to axial flow, we showed that flow profiles are not an independent predictor of vasoplegia. In contrast to flow profiles, previous cardiothoracic surgery, the use of angiotensin II receptor blockers, preoperative bilirubin and creatinine, and the use of fresh frozen plasma were found to be independent predictors for postoperative vasoplegia.

There is increasing evidence that differences in hemocompatibility, shear stress on the endothelial wall, and the release of pro- and anti-inflammatory cytokines may contribute to the development of postoperative vasoplegia [13, 21–24]. Axial flow (HMII) is thought to be less hemocompatible compared to centrifugal flow with a hybrid magnetic/hydrodynamic impeller (HVAD) and compared to centrifugal flow with an intrinsic artificial pulse with rapid changes in rotor speed (HM3), because of more degradation of von Willebrand factor high-molecular-weight multimers, low-grade hemolysis, and cytokine release. An increase in shear stress on the endothelial wall, caused by the continuous flow generated by the cfLVAD, causes a release of nitric oxide (NO) by endothelial signaling mechanisms with an effect on actin–myosin filaments contributing to vasodilation and thus contributing to the development of vasoplegia [13, 22]. This raised the question whether the unique flow profiles are related to the occurrence of postoperative vasoplegia. Although the artificial pulse rate provided by the HM3 is slower (maximum 30–40 per min) than and asynchronous with the innate heart rhythm, and with a different flow profile than that generated by cardiac ejection itself, systolic and diastolic blood pressures and pulse pressures can be measured or calculated [10, 11].

In our retrospective cohort, flow profiles were not an independent predictor, although there might be a tendency of a lower incidence of vasoplegia in patients with a continuous flow with artificial pulse device. However, several patient and procedural characteristics may carry more significance in the occurrence of postoperative vasoplegia than flow profiles, such as male gender, lower preoperative systolic and diastolic blood pressures, lower preoperative hemoglobin levels, higher bilirubin and creatine levels with a lower GFR, and the medical treatment of end-stage heart failure.

Firstly, in this retrospective cohort, axial flow cfLVADs (HMII) were implanted from 2006 to 2016 (Figure 1b), the implantation of centrifugal flow cfLVADs (HVAD) started in 2010 in our hospital, and the first centrifugal flow with artificial pulse cfLVAD (HM3) implantation was in 2015, and both latter devices were implanted until the end of the inclusions. The size of the axial flow pump requires opening of the peritoneum for its implantation. The newer LVADs are smaller, and the extent of the implantation procedure is less. Together with an improved surgical experience and improved surgical techniques and perioperative care, this must have an impact on the extent and duration of the operation, perioperative bleeding, use of blood products, and the possibility of reoperation for tamponade and/or bleeding probably having a role in better early and long-term outcomes after cfLVAD implantation.

Secondly, the medical treatment of end-stage chronic heart failure has undergone changes in the past with the more frequent use of newer drugs, such as angiotensin II receptor blockers —sometimes in combination with sacubitril — instead of ACE inhibitors, and the more frequent use of aldosterone antagonists in more recently implanted patients. ACE inhibitors and angiotensin II receptor blockers may possibly contribute to postoperative vasoplegia causing a lower systolic vascular resistance [25]. As shown in Table 1, patients implanted with an axial flow pump used significantly more often ACE inhibitors compared to the other patients (*p*=0.002). On the contrary, patients who received a centrifugal flow cfLVAD with(out) artificial pulse used more often angiotensin II receptor blockers (*p*=0.013) and aldosterone antagonists (*p*=0.034).

Thirdly, significantly more patients implanted with a centrifugal flow cfLVAD with or without artificial pulse were classified as INTERMACS IV to VI compared to patients who received an axial flow pump as these were more often classified as INTERMACS I and II. So, patients receiving a centrifugal flow cfLVAD were operated on earlier in their disease process, preventing a stage with sliding on inotropes or even worse. Moreover, it was reported that preoperative IL-6 and CRP levels were higher in INTERMACS I patients scheduled for cfLVAD implantation, suggesting that patients with end-stage heart failure classified in a lower class of INTERMACS score (e.g., INTERMACS I or II) probably have a more pronounced disbalance in inflammatory biomarkers preoperatively with a probable worse outcome, such as vasoplegia and related early post-cfLVAD implantation complications, such as right ventricular failure and kidney failure [26].

Fourthly, patients who received an axial flow pump had a statistically significant lower preoperative hemoglobin level probably necessitating significantly more perioperative blood transfusion compared to patients who received a centrifugal flow cfLVAD with(out) artificial pulse. This higher demand of blood transfusion is thought to cause a release of cytokines such as TNF-α, IL-1, and TNF-γ. The longer red blood cell units are stored, the higher is the level of inflammatory cytokines in these units [27]. These cytokines might contribute to a release of NO which is a factor in the development of vasoplegia [23, 28].

Finally, there are still several unknown factors that might have an effect on the incidence of cardiac vasoplegia syndrome in these patients, such as the use or not use of corticosteroids, the use of pulsatile flow profiles during extracorporeal circulation, and the use of cell saving techniques. These factors may have an impact on changes in inflammatory biomarkers and postoperative vasoplegia.

In summary, postoperative cardiac vasoplegia is a complex process provoked by many factors: preoperative factors, intraoperative factors, and postoperative factors. In end-stage heart failure, important preoperative factors, such as the severity of end-stage heart failure (INTERMACS class) with concomitant liver and kidney failure, the use of vasodilatory drugs, previous cardiothoracic surgery, intraoperative factors, such as used medications, the duration of extracorporeal circulation and the administration of blood products, and postoperative factors, such as LVAD flow profiles and postoperative bleeding, all play a crucial role in this complex nature of cardiac vasoplegia syndrome. All these factors more or less come together sometimes resulting in early (eventually intraoperatively) cardiac vasoplegia syndrome or late cardiac vasoplegia syndrome in the ICU. This may have a concomitant effect on further organ failure with most often worse outcomes.

Patients with postoperative vasoplegia suffered from more postoperative complications compared to patients without postoperative vasoplegia. Affected patients were significantly longer mechanically ventilated, developed more often renal failure and right ventricular failure, and more often major bleedings and cardiac tamponade (supplemental table 5a). Moreover, patients with a bad RV function preoperatively had a higher chance of RV failure postoperatively in the vasoplegia group. Patients with vasoplegia have a lower blood pressure despite higher vasopressor dosages, with probably a higher chance on lower right coronary artery blood flow and thus a higher chance to develop postoperative RV failure.

Regarding longer term outcomes, patients with postoperative vasoplegia had a longer ICU and hospital length of stay and a higher ICU and in-hospital mortality (supplemental table 5a). Moreover, ICU length of stay, hospital length of stay, and 1-year mortality improved from axial flow to centrifugal flow to centrifugal flow with intrinsic artificial pulse, while there was no difference observed in ICU, in-hospital, and 30-day mortality (supplemental table 5b). It is uncertain if the flow profile itself counts for this improved outcome or that it is caused by the abovementioned pre- and intraoperative factors.

### Limitations

We evaluated the impact of the various flow profile cfLVADs on vasoplegia and related early postoperative outcomes. However, several limitations exist. First, some of the variables used for analysis were incomplete. The maximum missingness percentage was less than 3% per variable (supplemental table 1), and therefore, the bias is minimal. We used multiple imputations to deal with the missing data, so no patients were excluded for analysis. Due to the retrospective nature of the study, it was necessary to adjust for other important variables. An extensive set of clinical variables were included for analysis. Therefore, LASSO logistic regression was used to reduce the number of variables. However, possibly other unmeasured or unknown factors may have affected the risk of vasoplegia and thus outcomes.

### Future Perspectives

Since both axial flow and the centrifugal flow cfLVADs are no longer available on the market, a prospective study in a larger multicenter study to evaluate the effect of axial and centrifugal flow profiles on vasoplegia itself and many confounders is no longer indicated. A deeper knowledge of the effect of flow profiles on vasoplegia may contribute to the development of newer cfLVADs, such as the Cleveland clinic continuous-flow total artificial heart (CFTAH) generating pulsatile flows by speed modulation in continuous-flow pumps [29, 30], the CorWave [31] (CorWave SA, Clichy, France), the Aeson TAH [32] (Carmat SA, Velizy-Villacoublay, France), Icoms flowmaker [33] (FineHeart, Sorigny, France), and EVAHEART®2 left ventricular assist device (EVA2) [34] (Evaheart Inc, Bellaire, TX, USA). It was suggested that pulsatility imitates the normal physiologic circulation with mechanical energy transmission to the vascular endothelium influencing cyclic endothelial shear stress and release of vasoactive molecules with improved end-organ microcirculation. Identifying perioperative specific biomarkers and cytokines in patients scheduled for cfLVAD implantation with different cfLVAD flow profiles may be of interest to assess the added value of pulsatility on the microcirculation.

## Conclusions

Although the incidence of vasoplegia decreased over time with the use of newer types of LVADs, flow profile in cfLVADs was not an independent risk factor for the development of postoperative vasoplegia. Several patient and procedural characteristics may carry more significance in the occurrence of postoperative vasoplegia than flow profiles, such as male gender, lower preoperative systolic and diastolic blood pressures, and lower preoperative hemoglobin levels. Moreover, previous cardiothoracic surgery, preoperative use of angiotensin II receptor blockers, preoperative bilirubin and creatinine levels, and the perioperative use of fresh frozen plasma were independent predictors of postoperative vasoplegia. Furthermore, this was associated with longer postoperative ventilation, higher incidence of major bleeding, tamponade, renal failure, right ventricular failure, longer ICU and hospital length of stay, and higher ICU mortality, hospital mortality, and 1-year mortality.

### Supplementary Information

ESM 1 (DOCX 15 kb)

ESM 2 (DOCX 31 kb)

ESM 3 (DOCX 21 kb)

ESM 4 (DOCX 20 kb)

ESM 5 (DOCX 33 kb)

## Abbreviations

*cfLVAD*: continuous-flow left ventricular assist device
*CI*: cardiac index
*FFP*: fresh frozen plasma
*Hb*: hemoglobin
*HMII*: HeartMate II
*HM3*: HeartMate 3
*HVAD*: HeartWare ventricular assist device
*ICU*: intensive care unit
*INTERMACS*: Interagency Registry for Mechanically Assisted Circulatory Support
*LOS*: length of stay
*LVAD*: left ventricular assist device
*MAP*: mean arterial pressure
*NO*: nitric oxide
*RBC*: red blood cells
*RVAD*: right ventricular assist device
*SVR*: systemic vascular resistance
