# Supplementary material for: FLAVOUR Study: FLow profiles And postoperative VasOplegia after continUous-flow left ventriculaR assist device implantation
Source: J Cardiovasc Transl Res. 2024 Feb 1;17(2):252–64. doi: 10.1007/s12265-023-10476-5 (PMC11052811; doi:10.1007/s12265-023-10476-5)
Supplement: Supplementary file 1 — (DOCX 15 kb) [file 12265_2023_10476_MOESM1_ESM.docx]

**Supplemental table 1:** Missing data percentage

|  |  |  |  |  | Missing data (%) |  |
| --- | --- | --- | --- | --- | --- | --- |
| Baseline characteristics | | | |  |  |  |
|  | Age (years) | |  |  | 0 |  |
|  | Male sex | |  |  | 0 |  |
|  | Weight (kg) | |  |  | 0 |  |
|  | BSA (m2) | |  |  | 0 |  |
|  | BMI (kg/m2) | |  |  | 0 |  |
|  | Systolic blood pressure (mmHg) | | |  | 1.0 |  |
|  | Diastolic blood pressure (mmHg) | | |  | 1.0 |  |
|  | Hypertension | |  |  | 12.8 |  |
|  | Smoking History | |  |  | 0 |  |
|  | COPD/CARA | |  |  | 0 |  |
|  | Previous Cardiothoracic Surgery | | |  | 0 |  |
|  | **Pre-operative medication** | | |  |  |  |
|  |  | Bèta blocker | |  | 0 |  |
|  |  | ACE-inhibitor | |  | 0 |  |
|  |  | ATII-receptor antagonist | |  | 0 |  |
|  |  | Lis-diuretics | |  | 0 |  |
|  |  | Aldosteron antagonist | |  | 0 |  |
|  |  | Dopamine | |  | 0 |  |
|  |  | Dobutamine | |  | 0 |  |
|  |  | Milrinone | |  | 0 |  |
|  |  | Noradrenaline | |  | 0 |  |
|  | **Pre-operative blood values** | | |  |  |  |
|  |  | Hemoglobin (mmol/L) | |  | 0.7 |  |
|  |  | Bilirubin (umol/L) | |  | 1.0 |  |
|  |  | ASAT (U/L) | |  | 2.1 |  |
|  |  | ALAT (U/L) | |  | 1.0 |  |
|  |  | Creatinin (umol/L) | |  | 0.3 |  |
|  |  | GFR (mL/min) | |  | 0.7 |  |
|  | Type of heart failure | | |  | 0 |  |
|  | INTERMACS classification | | |  | 0 |  |
|  | Right ventricle function | | |  | 1.0 |  |
|  | **Intraoperative management** | | |  |  |  |
|  |  | Skin-to-skin time (min) | |  | 0.7 |  |
|  |  | Bypass time (min) | |  | 0 |  |
|  |  | Red blood cells (units) | |  | 0 |  |
|  |  | Thrombocytes (units) | |  | 0 |  |
|  |  | Fresh Frozen Plasma (units) | |  | 0 |  |
|  |  | Total units blood | |  | 0 |  |
|  |  | Cell Saver (mL) | |  | 0.3 |  |
|  |  | Milrinone | |  | 0 |  |
|  |  | Dobutamine | |  | 0 |  |
|  |  | Noradrenalin | |  | 0 |  |
|  |  | Dopamine | |  | 0.3 |  |
|  |  | NO | |  | 0 |  |
