# Supplementary material for: FLAVOUR Study: FLow profiles And postoperative VasOplegia after continUous-flow left ventriculaR assist device implantation
Source: J Cardiovasc Transl Res. 2024 Feb 1;17(2):252–64. doi: 10.1007/s12265-023-10476-5 (PMC11052811; doi:10.1007/s12265-023-10476-5)
Supplement: Supplementary file 2 — (DOCX 31 kb) [file 12265_2023_10476_MOESM2_ESM.docx]

**Supplemental table 2a, 2b and 2c**. Baseline characteristics of patients stratified to cfLVAD flow profiles and postoperative vasoplegia.

**Supplemental table 2a**.

| **Axial flow (n=122)** | | | |
| --- | --- | --- | --- |
|  | Vasoplegia (n=37) | No vasoplegia  (n=85) | P-value |
| **Demographic data** | | | |
| Age (years) | 50.4 ± 10.9 | 48.7 ± 13.5 | 0.49 |
| Male gender | 29 (78.4%) | 56 (65.9%) | 0.17 |
| Weight (kg) | 74.2 ± 13.4 | 74.4 ± 14.6 | 0.94 |
| BSA (m2) | 1.92 ± 0.19 | 1.90 ± 0.21 | 0.65 |
| BMI (kg/m2) | 23.2 ± 3.5 | 23.9 ± 4.2 | 0.16 |
| **Patient History** | | | |
| Systolic blood pressure (mmHg) | 94 ± 16 | 101 ± 14 | <0.01 |
| Diastolic blood pressure (mmHg) | 60 ± 9 | 64 ± 11 | 0.05 |
| Smoking History | 20 (54.1%) | 40 (47.1%) | 0.48 |
| COPD/CARA | 2 (5.4%) | 15 (17.6%) | n.s. |
| Previous Cardiothoracic Surgery | 11 (29.7%) | 7 (8.2%) | <0.01 |
| **Pre-operative medication** | | | |
| LMWH | 2 (5.4%) | 22 (25.9%) | n.s. |
| Bèta blocker | 12 (32.4%) | 30 (35.3%) | 0.76 |
| ACE-inhibitor | 14 (37.8%) | 38 (44.7%) | 0.48 |
| ATII-receptor antagonist | 7 (18.9%) | 8 (9.4%) | 0.14 |
| Loop diuretics | 36 (97.3%) | 76 (89.4%) | 0.14 |
| Aldosteron antagonist | 23 (62.2%) | 62 (72.9%) | 0.23 |
| Dopamine | 10 (27.0%) | 13 (15.3%) | 0.13 |
| Dobutamine | 25 (67.6%) | 49 (57.6%) | 0.30 |
| Milrinone | 18 (48.6%) | 34 (40.0%) | 0.38 |
| Noradrenaline | 3 (8.1%) | 1 (1.2%) | n.s. |
| **Pre-operative laboratory data** | | | |
| Hemoglobin (mmol/L) | 7.5 ± 1.1 | 7.8 ± 1.2 | 0.15 |
| Bilirubin (umol/L) | 32 [23 – 43] | 23 [16 – 38] | 0.01 |
| ASAT (U/L) | 36 [27 – 85] | 36 [26 – 66] | 0.64 |
| ALAT (U/L) | 44 [26 – 163] | 49 [27 – 105] | 0.81 |
| Creatinin (umol/L) | 149 ± 52 | 114 ± 35 | <0.01 |
| GFR | 52 ± 23 | 66 ± 22 | <0.01 |
| **Type of heart failure** | | | |
| Ischemic | 13 (35.1%) | 11 (12.9%) | <0.01 |
| Dilated | 21 (56.8%) | 56 (65.9%) | 0.34 |
| Myocarditis | 0 (0.0%) | 8 (9.4%) | n.s. |
| Peri-partum | 0 (0.0%) | 3 (3.5%) | n.s. |
| Hypertrophic | 2 (5.4%) | 2 (2.4%) | n.s. |
| Toxic | 1 (2.7%) | 5 (5.9%) | n.s. |
| **Right ventricular function** | | | |
| Good | 5 (13.5%) | 17 (20.0%) | 0.28 |
| Moderate | 24 (64.9%) | 49 (57.6%) | 0.28 |
| Bad | 7 (18.9%) | 17 (20.0%) | 0.56 |
| **INTERMACS classification** | | | |
| INTERMACS Class I | 3 (8.1%) | 1 (1.2%) | n.s. |
| INTERMACS Class II | 19 (51.4%) | 48 (56.5%) | 0.37 |
| INTERMACS Class III | 13 (35.1%) | 3 (35.3%) | n.s. |
| INTERMACS Class IV - VI | 2 (5.4%) | 6 (7.0%) | n.s. |

n.s.: no statistics performed (for example because of too little numbers)

**Supplemental table 2b**.

| **Centrifugal flow (n=72)** | | | |
| --- | --- | --- | --- |
|  | Vasoplegia (n=18) | No vasoplegia  (n=54) | P-value |
| **Demographic data** | | | |
| Age (years) | 57.1 ± 12.8 | 53.8 ± 12.9 | 0.34 |
| Male gender | 15 (83.3%) | 30 (55.6%) | 0.04 |
| Weight (kg) | 75.5 ± 13.2 | 74.0 ± 13.9 | 0.70 |
| BSA (m2) | 1.92 ± 0.18 | 1.89 ± 0.19 | 0.46 |
| BMI (kg/m2) | 23.9 ± 3.6 | 24.2 ± 4.0 | 0.75 |
| **Patient History** | | | |
| Systolic blood pressure (mmHg) | 97 ± 13 | 104 ± 15 | 0.07 |
| Diastolic blood pressure (mmHg) | 64 ± 7 | 69 ± 13 | 0.10 |
| Smoking History | 6 (33.3%) | 17 (31.5%) | 0.89 |
| COPD/CARA | 3 (16.7%) | 17 (31.5%) | n.s. |
| Previous Cardiothoracic Surgery | 5 (27.8%) | 7 (13.0%) | 0.14 |
| **Pre-operative medication** | | | |
| LMWH | 5 (27.8%) | 23 (42.6%) | 0.26 |
| Bèta blocker | 4 (22.2%) | 14 (25.9%) | n.s. |
| ACE-inhibitor | 2 (11.1%) | 23 (42.6%) | n.s. |
| ATII-receptor antagonist | 4 (22.2%) | 7 (13.0%) | 0.34 |
| Loop diuretics | 15 (83.3%) | 48 (88.9%) | 0.54 |
| Aldosteron antagonist | 14 (77.8%) | 45 (83.3%) | 0.60 |
| Dopamine | 2 (11.1%) | 0 (0.0%) | n.s. |
| Dobutamine | 6 (33.3%) | 29 (53.7%) | 0.13 |
| Milrinone | 8 (44.4%) | 18 (33.3%) | 0.40 |
| Noradrenaline | 1 (5.6%) | 3 (5.6%) | n.s. |
| **Pre-operative laboratory data** | | | |
| Hemoglobin (mmol/L) | 7.8 ± 1.3 | 8.3 ± 1.2 | 0.17 |
| Bilirubin (umol/L) | 28 [25 – 33] | 17 [12 – 30] | 0.02 |
| ASAT (U/L) | 33 [24 – 60] | 34 [26 – 53] | 0.96 |
| ALAT (U/L) | 47 [28 – 96] | 38 [24 – 61] | 0.27 |
| Creatinin (umol/L) | 167 ± 75 | 106 ± 37 | <0.01 |
| GFR | 46 ± 20 | 69 ± 27 | <0.01 |
| **Type of heart failure** | | | |
| Ischemic | 8 (44.4%) | 15 (27.8%) | 0.19 |
| Dilated | 9 (50.0%) | 35 (64.8%) | 0.26 |
| Myocarditis | 0 (0.0%) | 1 (1.9%) | n.s. |
| Peri-partum | 0 (0.0%) | 1 (1.9%) | n.s. |
| Hypertrophic | 1 (5.6%) | 1 (1.9%) | n.s. |
| Toxic | 0 (0.0%) | 1 (1.9%) | n.s. |
| **Right ventricular function** | | | |
| Good | 7 (38.9%) | 16 (29.6%) | 0.33 |
| Moderate | 10 (55.6%) | 32 (59.3%) | 0.50 |
| Bad | 1 (5.6%) | 6 (11.1%) | n.s. |
| **INTERMACS classification** | | | |
| INTERMACS Class I | 0 (0.0%) | 3 (5.6%) | n.s. |
| INTERMACS Class II | 9 (50.0%) | 15 (27.8%) | 0.07 |
| INTERMACS Class III | 4 (22.2%) | 25 (46.3%) | n.s. |
| INTERMACS Class IV - VI | 5 (27.8%) | 11 (20.4%) | n.s. |

n.s.: no statistics performed (for example because of too little numbers)

**Supplemental table 2c**.

| **Centrifugal flow with intrinsic pulse (n=95)** | | | |
| --- | --- | --- | --- |
|  | Vasoplegia (n=18) | No vasoplegia  (n=77) | P-value |
| **Demographic data** | | | |
| Age (years) | 58.1 ± 10.3 | 51.0 ± 14.0 | 0.05 |
| Male gender | 15 (83.3%) | 48 (62.3%) | 0.09 |
| Weight (kg) | 84.4 ± 12.1 | 76.4 ± 14.7 | 0.03 |
| BSA (m2) | 2.04 ± 0.17 | 1.93 ± 0.21 | 0.06 |
| BMI (kg/m2) | 26.1 ± 2.3 | 24.2 ± 4.2 | 0.07 |
| **Patient History** | | | |
| Systolic blood pressure (mmHg) | 97 ± 13 | 104 ± 15 | 0.07 |
| Diastolic blood pressure (mmHg) | 64 ± 7 | 69 ± 13 | 0.10 |
| Smoking History | 8 (44.4%) | 32 (41.6%) | 0.51 |
| COPD/CARA | 1 (5.6%) | 8 (10.4%) | n.s. |
| Previous Cardiothoracic Surgery | 4 (22.2%) | 7 (9.1%) | n.s. |
| **Pre-operative medication** | | | |
| LMWH | 12 (66.7%) | 43 (55.8%) | 0.40 |
| Bèta blocker | 2 (11.1%) | 15 (19.5%) | n.s. |
| ACE-inhibitor | 1 (5.6%) | 18 (23.4%) | n.s. |
| ATII-receptor antagonist | 8 (44.4%) | 18 (23.4%) | 0.07 |
| Loop diuretics | 18 (100.0%) | 69 (89.6%) | 0.15 |
| Aldosteron antagonist | 15 (83.3%) | 64 (83.1%) | 0.98 |
| Dopamine | 0 (0.0%) | 4 (5.2%) | n.s. |
| Dobutamine | 10 (55.6%) | 35 (45.5%) | 0.44 |
| Milrinone | 6 (33.3%) | 29 (37.7%) | 0.73 |
| Noradrenaline | 2 (11.1%) | 5 (6.5%) | n.s. |
| **Pre-operative laboratory data** | | | |
| Hemoglobin (mmol/L) | 8.2 ± 1.1 | 8.4 ± 1.2 | 0.53 |
| Bilirubin (umol/L) | 28 [14 – 32] | 16 [10 – 30] | 0.11 |
| ASAT (U/L) | 34 [24 – 46] | 35 [22 – 49] | 0.99 |
| ALAT (U/L) | 35 [25 – 62] | 48 [24 – 117] | 0.33 |
| Creatinin (umol/L) | 131 ± 50 | 111 ± 42 | 0.07 |
| GFR | 58 ± 25 | 70 ± 29 | 0.11 |
| **Type of heart failure** | | | |
| Ischemic | 6 (33.3%) | 12 (18.2%) | 0.16 |
| Dilated | 12 (66.7%) | 58 (75.3%) | 0.45 |
| Myocarditis | 0 (0.0%) | 1 (1.3%) | n.s. |
| Peri-partum | 0 (0.0%) | 1 (1.3%) | n.s. |
| Hypertrophic | 0 (0.0%) | 1 (1.3%) | n.s. |
| Toxic | 0 (0.0%) | 2 (2.6%) | n.s. |
| **Right ventricular function** | | | |
| Good | 4 (22.2%) | 31 (40.3%) | n.s. |
| Moderate | 12 (66.7%) | 36 (46.8%) | 0.10 |
| Bad | 2 (11.1%) | 10 (13.0%) | n.s. |
| **INTERMACS classification** | | | |
| INTERMACS Class I | 1 (5.6%) | 3 (3.9%) | n.s. |
| INTERMACS Class II | 7 (38.9%) | 27 (35.1%) | 0.48 |
| INTERMACS Class III | 7 (38.9%) | 26 (33.8%) | 0.44 |
| INTERMACS Class IV - VI | 3 (16.7%) | 21 (27.3%) | n.s. |

n.s.: no statistics performed (for example because of too little numbers)
