# Supplementary material for: FLAVOUR Study: FLow profiles And postoperative VasOplegia after continUous-flow left ventriculaR assist device implantation
Source: J Cardiovasc Transl Res. 2024 Feb 1;17(2):252–64. doi: 10.1007/s12265-023-10476-5 (PMC11052811; doi:10.1007/s12265-023-10476-5)
Supplement: Supplementary file 3 — (DOCX 21 kb) [file 12265_2023_10476_MOESM3_ESM.docx]

**Supplemental table 3.** Postoperative use of inotropes and vasopressors stratified to flow profiles

|  | Axial flow (n=122) | Centrifugal flow (n=72) | Centrifugal flow with artificial pulse (n=95) | P-value |
| --- | --- | --- | --- | --- |
| **Mean dosage inotropes and vasopressors** | | | | |
| Noradrenaline 0-24h (ng/kg/min) | 255 ± 228 | 194 ± 186 | 178 ± 142 | 0.01 |
| Noradrenaline 24-48h (ng/kg/min) | 147 ± 232 | 114 ± 179 | 77 ± 106 | 0.02 |
| Noradrenaline 0-48h (ng/kg/min) | 201 ± 218 | 154 ± 173 | 128 ± 115 | 0.01 |
| Dobutamine 0-24h (mcg/kg/min) | 4.6 ± 3.2 | 3.9 ± 2.0 | 3.7 ± 2.3 | 0.02 |
| Dobutamine 24-48h (mcg/kg/min) | 3.4 ± 2.9 | 2.9 ± 2.3 | 2.4 ± 2.1 | 0.03 |
| Dobutamine 0-48h (mcg/kg/min) | 4.0 ± 2.9 | 3.4 ± 2.0 | 3.1 ± 2.0 | 0.02 |
| Dopamine 0-24h (mcg/kg/min) | 0.6 ± 1.7 | 0.0 ± 0.0 | 0.0 ± 0.0 | n.s. |
| Dopamine 24-48h (mcg/kg/min) | 0.4 ± 1.3 | 0.0 ± 0.0 | 0.0 ± 0.0 | n.s. |
| Dopamine 0-48h (mcg/kg/min) | 0.5 ± 1.5 | 0.0 ± 0.0 | 0.0 ± 0.0 | n.s. |
| Milrinone 0-24h (mcg/kg/min) | 0.23 ± 0.12 | 0.19 ± 0.13 | 0.17 ± 0.10 | <0.01 |
| Milrinone 24-48h (mcg/kg/min) | 0.16 ± 0.14 | 0.13 ± 0.13 | 0.12 ± 0.11 | 0.08 |
| Milrinone 0-48h (mcg/kg/min) | 0.19 ± 0.13 | 0.16 ± 0.12 | 0.14 ± 0.10 | <0.01 |
| Vasopressin 0-24h (IU/h) | 0.2 ± 0.7 | 0.3 ± 0.7 | 0.6 ± 1.0 | <0.01 |
| Vasopressin 24-48h (IU/h) | 0.1 ± 0.5 | 0.1 ± 0.6 | 0.2 ± 0.7 | 0.48 |
| Vasopressin 0-48h (IU/h) | 0.2 ± 0.6 | 0.2 ± 0.6 | 0.4 ± 0.7 | 0.02 |
| Epinephrin 0-24h (mcg/kg/min) | 0.004 ± 0.030 | 0.002 ± 0.010 | 0.001 ± 0.008 | 0.46 |
| Epinephrin 24-48h (mcg/kg/min) | 0.002 ± 0.018 | 0.000 ±0.002 | 0.001 ± 0.006 | 0.60 |
| Epinephrin 0-48h (mcg/kg/min) | 0.003 ± 0.023 | 0.001 ± 0.006 | 0.001 ± 0.005 | 0.49 |
| **Duration of use of inotropes and vasopressors** | | | | |
| Noradrenaline 0h-1h | 5 (4.1%) | 1 (1.4%) | 0 (0.0%) | n.s. |
| Noradrenaline 1h-24h | 38 (31.1%) | 18 (25.0%) | 36 (37.9%) | 0.20 |
| Noradrenaline 25h-48h | 79 (64.8%) | 53 (73.6%) | 59 (62.1%) | 0.27 |
| Dobutamine 0h-1h | 17 (13.9%) | 1 (1.4%) | 11 (11.6%) | n.s. |
| Dobutamine 1h-24h | 13 (10.7%) | 11 (15.3%) | 18 (18.9%) | 0.22 |
| Dobutamine 25h-48h | 92 (75.4%) | 60 (83.3%) | 66 (69.5%) | 0.12 |
| Dopamine 0h-1h | 196 (86.9%) | 72 (100%) | 95 (100%) | <0.01 |
| Dopamine 1h-24h | 5 (4.1%) | 0 (0.0%) | 0 (0.0%) | n.s. |
| Dopamine 25h-48h | 11 (9.0%) | 0 (0.0%) | 0 (0.0%) | n.s. |
| Milrinone 0h-1h | 5 (4.1%) | 10 (13.9%) | 6 (6.3%) | 0.04 |
| Milrinone 1h-24h | 28 (23.0%) | 15 (20.8%) | 22 (23.2%) | 0.93 |
| Milrinone 25h-48h | 89 (73.0%) | 47 (65.3%) | 67 (70.5%) | 0.53 |
| Vasopressin 0h-1h | 106 (86.9%) | 47 (65.3%) | 51 (53.7%) | <0.01 |
| Vasopressin 1h-24h | 8 (6.6%) | 18 (25.0%) | 29 (30.5%) | <0.01 |
| Vasopressin 25h-48h | 8 (6.6%) | 7 (9.7%) | 15 (15.8%) | 0.09 |
| Epinephrin 0h-1h | 117 (95.9%) | 68 (94.4%) | 92 (96.8%) | 0.74 |
| Epinephrin 1h-24h | 3 (2.5%) | 3 (4.2%) | 3 (3.2%) | n.s. |
| Epinephrin 25h-48h | 2 (1.6%) | 1 (1.4%) | 0 (0.0%) | n.s. |
| **Number of inotropes used (dopamine, dobutamine, milrinone)** | | | | |
| 0 inotropes | 1 (0.8%) | 0 (0.0%) | 0 (0.0%) | n.s. |
| 1 inotrope | 10 (8.2%) | 11 (15.3%) | 17 (17.9%) | 0.09 |
| 2 inotropes | 105 (86.1%) | 61 (84.7%) | 78 (82.1%) | 0.73 |
| 3 inotropes | 6 (4.9%) | 0 (0.0%) | 0 (0.0%) | n.s. |
| **Number of vasopressors used (noradrenaline, vasopressin, epinephrin)** | | | | |
| 0 vasopressors | 5 (4.1%) | 1 (1.4%) | 0 (0.0%) | n.s. |
| 1 vasopressor | 97 (79.5%) | 46 (63.9%) | 51 (53.7%) | <0.01 |
| 2 vasopressors | 19 (15.6%) | 21 (29.2%) | 41 (43.2%) | <0.01 |
| 3 vasopressors | 1 (0.8%) | 4 (5.6%) | 3 (3.2%) | n.s. |

n.s.: no statistics performed (for example because of too little numbers)
