# Supplementary material for: FLAVOUR Study: FLow profiles And postoperative VasOplegia after continUous-flow left ventriculaR assist device implantation
Source: J Cardiovasc Transl Res. 2024 Feb 1;17(2):252–64. doi: 10.1007/s12265-023-10476-5 (PMC11052811; doi:10.1007/s12265-023-10476-5)
Supplement: Supplementary file 4 — (DOCX 20 kb) [file 12265_2023_10476_MOESM4_ESM.docx]

**Supplemental table 4.** Postoperative use of inotropes and vasopressors stratified to vasoplegia

|  | Vasoplegia (n=73) | No vasoplegia (n=216) | P-value | |
| --- | --- | --- | --- | --- |
| **Mean dosage inotropes and vasopressors** | | | | |
| Noradrenaline 0-24h (ng/kg/min) | 401 ± 202 | 151 ± 147 | <0.01 | |
| Noradrenaline 24-48h (ng/kg/min) | 258 ± 273 | 68 ± 114 | <0.01 | |
| Noradrenaline 0-48h (ng/kg/min) | 329 ± 225 | 110 ± 121 | <0.01 | |
| Dobutamine 0-24h (mcg/kg/min) | 4.4 ± 3.1 | 4.1 ± 2.5 | 0.39 | |
| Dobutamine 24-48h (mcg/kg/min) | 3.0 ± 2.8 | 2.9 ± 2.4 | 0.85 | |
| Dobutamine 0-48h (mcg/kg/min) | 3.7 ± 2.8 | 3.5 ± 2.3 | 0.58 | |
| Dopamine 0-24h (mcg/kg/min) | 0.4 ± 1.2 | 0.2 ± 1.1 | 0.22 | |
| Dopamine 24-48h (mcg/kg/min) | 0.2 ± 0.8 | 0.2 ± 0.9 | 0.71 | |
| Dopamine 0-48h (mcg/kg/min) | 0.3 ± 1.0 | 0.2 ± 1.0 | 0.39 | |
| Milrinone 0-24h (mcg/kg/min) | 0.21 ± 0.14 | 0.19 ± 0.11 | 0.30 | |
| Milrinone 24-48h (mcg/kg/min) | 0.15 ± 0.14 | 0.14 ± 0.13 | 0.78 | |
| Milrinone 0-48h (mcg/kg/min) | 0.18 ± 0.13 | 0.17 ± 0.11 | 0.50 | |
| Vasopressin 0-24h (IU/h) | 0.8 ± 1.1 | 0.2 ± 0.7 | <0.01 | |
| Vasopressin 24-48h (IU/h) | 0.3 ± 0.8 | 0.1 ± 0.5 | <0.01 | |
| Vasopressin 0-48h (IU/h) | 0.6 ± 0.9 | 0.2 ± 0.5 | <0.01 | |
| Epinephrin 0-24h (mcg/kg/min) | 0.008 ± 0.039 | 0.001 ± 0.007 | <0.01 | |
| Epinephrin 24-48h (mcg/kg/min) | 0.003 ± 0.024 | 0.000 ± 0.004 | 0.09 | |
| Epinephrin 0-48h (mcg/kg/min) | 0.006 ± 0.030 | 0.001 ± 0.005 | 0.02 | |
| **Duration of use of inotropes and vasopressors** | | | | |
| Noradrenaline 0h-1h | 0 (0.0%) | 6 (2.8%) | n.s. | |
| Noradrenaline 1h-24h | 5 (6.8%) | 87 (40.3%) | <0.01 | |
| Noradrenaline 25h-48h | 68 (83.2%) | 123 (56.9%) | <0.01 | |
| Dobutamine 0h-1h | 8 (11.0%) | 21 (9.7%) | 0.76 | |
| Dobutamine 1h-24h | 10 (13.7%) | 32 (14.8%) | 0.82 | |
| Dobutamine 25h-48h | 55 (75.3%) | 163 (75.5%) | 0.98 | |
| Dopamine 0h-1h | 65 (89.0%) | 208 (96.3%) | 0.02 | |
| Dopamine 1h-24h | 3 (4.1%) | 2 (0.9%) | n.s. | |
| Dopamine 25h-48h | 5 (6.8%) | 6 (2.8%) | 0.12 | |
| Milrinone 0h-1h | 4 (5.5%) | 17 (7.9%) | n.s. | |
| Milrinone 1h-24h | 18 (24.7%) | 47 (21.8%) | 0.61 | |
| Milrinone 25h-48h | 51 (69.9%) | 152 (70.4%) | 0.94 | |
| Vasopressin 0h-1h | 34 (46.6%) | 170 (78.7%) | <0.01 | |
| Vasopressin 1h-24h | 24 (32.9%) | 31 (14.4%) | <0.01 | |
| Vasopressin 25h-48h | 15 (20.5%) | 15 (6.9%) | <0.01 | |
| Epinephrin 0h-1h | 66 (90.4%) | 211 (97.7%) | <0.01 | |
| Epinephrin 1h-24h | 5 (6.8%) | 4 (1.9%) | 0.03 | |
| Epinephrin 25h-48h | 2 (2.7%) | 1 (0.5%) | n.s. | |
| **Number of inotropes used (dopamine, dobutamine, milrinone)** | | | | |
| 0 inotropes | 1 (1.4%) | 0 (0.0%) | n.s. | |
| 1 inotrope | 7 (9.6%) | 31 (14.4%) | 0.30 | |
| 2 inotropes | 60 (82.2%) | 184 (85.2%) | 0.54 | |
| 3 inotropes | 5 (6.8%) | 1 (0.5%) | n.s. | |
| **Number of vasopressors used (noradrenaline, vasopressin, epinephrin)** | | | | |
| 0 vasopressors | 0 (0.0%) | 6 (2.8%) | | n.s. |
| 1 vasopressor | 30 (41.1%) | 164 (75.9%) | | <0.01 |
| 2 vasopressors | 40 (54.8%) | 41 (19.0%) | | <0.01 |
| 3 vasopressors | 5 (2.3%) | 3 (4.1%) | | n.s. |

n.s.: no statistics performed (for example because of too little numbers)
