# Supplementary material for: FLAVOUR Study: FLow profiles And postoperative VasOplegia after continUous-flow left ventriculaR assist device implantation
Source: J Cardiovasc Transl Res. 2024 Feb 1;17(2):252–64. doi: 10.1007/s12265-023-10476-5 (PMC11052811; doi:10.1007/s12265-023-10476-5)
Supplement: Supplementary file 5 — (DOCX 33 kb) [file 12265_2023_10476_MOESM5_ESM.docx]

**Supplemental table 5a, 5b, 5c.** Postoperative use of inotropes and vasopressors stratified to flow profiles and vasoplegia.

**Supplemental table 5a.**

| **Axial flow (n=122)** | | | |
| --- | --- | --- | --- |
|  | Vasoplegia (n=37) | No vasoplegia (n=85) | P-value |
| **Mean dosage inotropes and vasopressors** | | | |
| Noradrenaline 0-24h (ng/kg/min) | 447 ± 230 | 171 ± 171 | <0.01 |
| Noradrenaline 24-48h (ng/kg/min) | 316 ± 318 | 74 ± 128 | <0.01 |
| Noradrenaline 0-48h (ng/kg/min) | 382 ± 260 | 123 ± 137 | <0.01 |
| Dobutamine 0-24h (mcg/kg/min) | 4.5 ± 3.7 | 4.7 ± 3.0 | 0.67 |
| Dobutamine 24-48h (mcg/kg/min) | 3.4 ± 3.0 | 3.3 ± 2.8 | 0.89 |
| Dobutamine 0-48h (mcg/kg/min) | 3.9 ± 3.2 | 4.0 ± 2.8 | 0.86 |
| Dopamine 0-24h (mcg/kg/min) | 0.7 ±1.6 | 0.5 ± 1.7 | 0.45 |
| Dopamine 24-48h (mcg/kg/min) | 0.4 ± 1.1 | 0.4 ± 1.5 | 0.99 |
| Dopamine 0-48h (mcg/kg/min) | 0.6 ± 1.3 | 0.4 ± 1.6 | 0.66 |
| Milrinone 0-24h (mcg/kg/min) | 0.24 ± 0.14 | 0.22 ± 0.11 | 0.57 |
| Milrinone 24-48h (mcg/kg/min) | 0.17 ± 0.15 | 0.16 ± 0.14 | 0.60 |
| Milrinone 0-48h (mcg/kg/min) | 0.20 ± 0.13 | 0.19 ± 0.12 | 0.57 |
| Vasopressin 0-24h (IU/h) | 0.5 ± 1.1 | 0.1 ± 0.4 | <0.01 |
| Vasopressin 24-48h (IU/h) | 0.3 ± 0.8 | 0.0 ± 0.2 | <0.01 |
| Vasopressin 0-48h (IU/h) | 0.4 ± 0.9 | 0.1 ± 0.3 | <0.01 |
| Epinephrin 0-24h (mcg/kg/min) | 0.015 ± 0.053 | 0 | n.s. |
| Epinephrin 24-48h (mcg/kg/min) | 0.007 ± 0.033 | 0 | n.s. |
| Epinephrin 0-48h (mcg/kg/min) | 0.011 ± 0.042 | 0 | n.s. |
| **Duration of use of inotropes and vasopressors** | | | |
| Noradrenaline 0h-1h | 0 (0.0%) | 5 (5.9%) | n.s. |
| Noradrenaline 1h-24h | 3 (8.1%) | 35 (41.2%) | <0.01 |
| Noradrenaline 25h-48h | 34 (91.0%) | 45 (52.9%) | <0.01 |
| Dobutamine 0h-1h | 5 (13.5%) | 12 (14.1%) | 0.93 |
| Dobutamine 1h-24h | 5 (13.5%) | 8 (9.4%) | 0.50 |
| Dobutamine 25h-48h | 27 (73.0%) | 65 (76.5%) | 0.68 |
| Dopamine 0h-1h | 29 (78.4%) | 77 (90.6%) | 0.07 |
| Dopamine 1h-24h | 3 (8.1%) | 2 (2.4%) | n.s. |
| Dopamine 25h-48h | 5 (13.5%) | 6 (7.1%) | 0.25 |
| Milrinone 0h-1h | 2 (5.4%) | 3 (3.5%) | n.s. |
| Milrinone 1h-24h | 8 (21.6%) | 20 (23.5%) | 0.82 |
| Milrinone 25h-48h | 27 (73.0%) | 62 (72.9%) | 0.99 |
| Vasopressin 0h-1h | 28 (75.7%) | 78 (91.8%) | 0.02 |
| Vasopressin 1h-24h | 4 (10.8%) | 4 (4.7%) | n.s. |
| Vasopressin 25h-48h | 5 (13.5%) | 3 (3.5%) | n.s. |
| Epinephrin 0h-1h | 32 (86.5%) | 85 (100.0%) | <0.01 |
| Epinephrin 1h-24h | 3 (8.1%) | 0 (0.0%) | n.s. |
| Epinephrin 25h-48h | 2 (5.4%) | 0 (0.0%) | n.s. |
| **Number of inotropes used (dopamine, dobutamine, milrinone)** | | | |
| 0 inotropes | 1 (2.7%) | 0 (0.0%) | n.s. |
| 1 inotrope | 2 (5.4%) | 8 (9.4%) | n.s. |
| 2 inotropes | 29 (78.4%) | 76 (89.4%) | 0.11 |
| 3 inotropes | 5 (13.5%) | 1 (1.2%) | n.s. |
| **Number of vasopressors used (noradrenaline, vasopressin, epinephrin)** | | | |
| 0 vasopressors | 0 (0.0%) | 5 (5.9%) | n.s. |
| 1 vasopressor | 24 (64.9%) | 73 (85.9%) | <0.01 |
| 2 vasopressors | 12 (32.4%) | 7 (8.2%) | <0.01 |
| 3 vasopressors | 1 (2.7%) | 0 (0.0%) | n.s. |

n.s.: no statistics performed (for example because of too little numbers)

**Supplemental table 5b.**

| **Centrifugal flow (n=72)** | | | |
| --- | --- | --- | --- |
|  | Vasoplegia (n=18) | No vasoplegia (n=54) | P-value |
| **Dosage inotropes and vasopressors** | | | |
| Noradrenaline 0-24h (ng/kg/min) | 376 ± 201 | 133 ± 135 | <0.01 |
| Noradrenaline 24-48h (ng/kg/min) | 229 ± 261 | 76 ± 123 | <0.01 |
| Noradrenaline 0-48h (ng/kg/min) | 303 ± 219 | 105 ± 122 | <0.01 |
| Dobutamine 0-24h (mcg/kg/min) | 4.7 ± 2.5 | 3.7 ± 1.7 | 0.05 |
| Dobutamine 24-48h (mcg/kg/min) | 3.1 ± 3.0 | 2.8 ± 2.0 | 0.60 |
| Dobutamine 0-48h (mcg/kg/min) | 3.9 ± 2.5 | 3.2 ± 1.7 | 0.19 |
| Dopamine 0-24h (mcg/kg/min) | 0 | 0 | n.s. |
| Dopamine 24-48h (mcg/kg/min) | 0 | 0 | n.s. |
| Dopamine 0-48h (mcg/kg/min) | 0 | 0 | n.s. |
| Milrinone 0-24h (mcg/kg/min) | 0.22 ± 0.16 | 0.18 ± 0.11 | 0.22 |
| Milrinone 24-48h (mcg/kg/min) | 0.16 ± 0.17 | 0.12 ± 0.12 | 0.29 |
| Milrinone 0-48h (mcg/kg/min) | 0.19 ± 0.16 | 0.15 ± 0.11 | 0.22 |
| Vasopressin 0-24h (IU/h) | 0.9 ± 0.9 | 0.2 ± 0.5 | <0.01 |
| Vasopressin 24-48h (IU/h) | 0.1 ± 0.2 | 0.1 ± 0.6 | 0.57 |
| Vasopressin 0-48h (IU/h) | 0.5 ± 0.5 | 0.2 ± 0.6 | 0.03 |
| Epinephrin 0-24h (mcg/kg/min) | 0.003 ± 0.010 | 0.001 ± 0.010 | 0.50 |
| Epinephrin 24-48h (mcg/kg/min) | 0 | 0.000 ± 0.003 | n.s. |
| Epinephrin 0-48h (mcg/kg/min) | 0.002 ± 0.005 | 0.001 ± 0.006 | 0.66 |
| **Duration of use of inotropes and vasopressors** | | | |
| Noradrenaline 0h-1h | 0 (0.0%) | 1 (1.9%) | n.s. |
| Noradrenaline 1h-24h | 0 (0.0%) | 18 (33.3%) | n.s. |
| Noradrenaline 25h-48h | 18 (100.0%) | 35 (64.8%) | <0.01 |
| Dobutamine 0h-1h | 0 (0.0%) | 1 (1.9%) | n.s. |
| Dobutamine 1h-24h | 2 (11.1%) | 9 (16.7%) | n.s. |
| Dobutamine 25h-48h | 16 (88.9%) | 44 (81.5%) | 0.47 |
| Dopamine 0h-1h | 18 (100.0%) | 54 (100.0%) | n.s. |
| Dopamine 1h-24h | 0 (0.0%) | 0 (0.0%) | n.s. |
| Dopamine 25h-48h | 0 (0.0%) | 0 (0.0%) | n.s. |
| Milrinone 0h-1h | 2 (11.1%) | 8 (14.8%) | n.s. |
| Milrinone 1h-24h | 4 (22.2%) | 11 (20.4%) | n.s. |
| Milrinone 25h-48h | 12 (66.7%) | 35 (64.8%) | 0.89 |
| Vasopressin 0h-1h | 5 (27.8%) | 42 (77.8%) | <0.01 |
| Vasopressin 1h-24h | 10 (55.6%) | 8 (14.8%) | <0.01 |
| Vasopressin 25h-48h | 3 (16.7%) | 4 (7.4%) | n.s. |
| Epinephrin 0h-1h | 16 (88.9%) | 52 (96.3%) | 0.24 |
| Epinephrin 1h-24h | 2 (11.1%) | 1 (1.9%) | n.s. |
| Epinephrin 25h-48h | 0 (0.0%) | 1 (1.9%) | n.s. |
| **Number of inotropes used (dopamine, dobutamine, milrinone)** | | | |
| 0 inotropes | 0 (0.0%) | 0 (0.0%) | n.s. |
| 1 inotrope | 2 (11.1%) | 9 (16.7%) | n.s. |
| 2 inotropes | 16 (88.9%) | 45 (83.3%) | 0.57 |
| 3 inotropes | 0 (0.0%) | 0 (0.0%) | n.s. |
| **Number of vasopressors used (noradrenaline, vasopresin, epinephrin)** | | | |
| 0 vasopressors | 0 (0.0%) | 1 (1.9%) | n.s. |
| 1 vasopressor | 5 (27.8%) | 41 (75.9%) | <0.01 |
| 2 vasopressors | 11 (61.1%) | 10 (18.5%) | <0.01 |
| 3 vasopressors | 2 (11.1%) | 2 (3.7%) | n.s. |

n.s.: no statistics performed (for example because of too little numbers)

**Supplemental table 5c.**

| **Centrifugal flow with artificial pulse (n=95)** | | | |
| --- | --- | --- | --- |
|  | Vasoplegia (n=18) | No vasoplegia (n=77) | P-value |
| **Dosage inotropes and vasopressors** | | | |
| Noradrenaline 0-24h (ng/kg/min) | 331 ± 103 | 142 ± 125 | <0.01 |
| Noradrenaline 24-48h (ng/kg/min) | 168 ± 130 | 56 ± 88 | <0.01 |
| Noradrenaline 0-48h (ng/kg/min) | 249 ± 97 | 99 ± 100 | <0.01 |
| Dobutamine 0-24h (mcg/kg/min) | 3.9 ± 2.3 | 3.6 ± 2.3 | 0.22 |
| Dobutamine 24-48h (mcg/kg/min) | 2.0 ± 1.9 | 2.6 ± 2.2 | 0.31 |
| Dobutamine 0-48h (mcg/kg/min) | 2.9 ± 1.9 | 3.1 ± 2.1 | 0.75 |
| Dopamine 0-24h (mcg/kg/min) | 0 | 0 | n.s. |
| Dopamine 24-48h (mcg/kg/min) | 0 | 0 | n.s. |
| Dopamine 0-48h (mcg/kg/min) | 0 | 0 | n.s. |
| Milrinone 0-24h (mcg/kg/min) | 0.14 ± 0.08 | 0.17 ± 0.10 | 0.27 |
| Milrinone 24-48h (mcg/kg/min) | 0.07 ± 0.07 | 0.13 ± 0.12 | 0.04 |
| Milrinone 0-48h (mcg/kg/min) | 0.11 ± 0.06 | 0.15 ± 0.10 | 0.09 |
| Vasopressin 0-24h (IU/h) | 1.3 ± 1.2 | 0.5 ± 0.9 | <0.01 |
| Vasopressin 24-48h (IU/h) | 0.5 ± 1.1 | 0.1 ± 0.5 | 0.03 |
| Vasopressin 0-48h (IU/h) | 0.9 ± 1.0 | 0.3 ± 0.6 | <0.01 |
| Epinephrin 0-24h (mcg/kg/min) | 0 | 0.001 ± 0.009 | n.s. |
| Epinephrin 24-48h (mcg/kg/min) | 0 | 0.001 ± 0.007 | n.s. |
| Epinephrin 0-48h (mcg/kg/min) | 0 | 0.001 ± 0.006 | n.s. |
| **Duration of use of inotropes and vasopressors** | | | |
| Noradrenaline 0h-1h | 0 (0.0%) | 0 (0.0%) | n.s. |
| Noradrenaline 1h-24h | 2 (11.1%) | 34 (44.2%) | n.s. |
| Noradrenaline 25h-48h | 16 (88.9%) | 43 (55.8%) | <0.01 |
| Dobutamine 0h-1h | 3 (16.7%) | 8 (10.4%) | n.s. |
| Dobutamine 1h-24h | 3 (16.7%) | 15 (19.5%) | n.s. |
| Dobutamine 25h-48h | 12 (66.7%) | 54 (70.1%) | 0.77 |
| Dopamine 0h-1h | 0 (0.0%) | 0 (0.0%) | n.s. |
| Dopamine 1h-24h | 0 (0.0%) | 0 (0.0%) | n.s. |
| Dopamine 25h-48h | 0 (0.0%) | 0 (0.0%) | n.s. |
| Milrinone 0h-1h | 0 (0.0%) | 6 (7.8%) | n.s. |
| Milrinone 1h-24h | 6 (33.3%) | 16 (20.8%) | 0.26 |
| Milrinone 25h-48h | 12 (66.7%) | 55 (71.4%) | 0.69 |
| Vasopressin 0h-1h | 1 (5.6%) | 50 (64.9%) | n.s. |
| Vasopressin 1h-24h | 10 (55.6%) | 19 (24.7%) | 0.01 |
| Vasopressin 25h-48h | 7 (38.9%) | 8 (10.4%) | <0.01 |
| Epinephrin 0h-1h | 18 (100.0%) | 74 (96.1%) | 0.40 |
| Epinephrin 1h-24h | 0 (0.0%) | 3 (3.9%) | n.s. |
| Epinephrin 25h-48h | 0 (0.0%) | 0 (0.0%) | n.s. |
| **Number of inotropes used (dopamine, dobutamine, milrinone)** | | | |
| 0 inotropes | 0 (0.0%) | 0 (0.0%) | n.s. |
| 1 inotrope | 3 (16.7%) | 14 (18.2%) | n.s. |
| 2 inotropes | 15 (83.3%) | 63 (81.8%) | 0.88 |
| 3 inotropes | 0 (0.0%) | 0 (0.0%) | n.s. |
| **Number of vasopressors used (noradrenaline, vasopressin, epinephrin)** | | | |
| 0 vasopressors | 0 (0.0%) | 0 (0.0%) | n.s. |
| 1 vasopressor | 1 (5.6%) | 50 (64.9%) | n.s. |
| 2 vasopressors | 17 (94.4%) | 24 (31.2%) | <0.01 |
| 3 vasopressors | 0 (0.0%) | 3 (3.9%) | n.s. |

n.s.: no statistics performed (for example because of too little numbers)
